# Correction: Androgen receptor promotes renal cell carcinoma (RCC) vasculogenic mimicry (VM) via altering TWIST1 nonsense-mediated decay through lncRNA-TANAR

**DOI:** 10.1038/s41388-025-03346-8

**Published:** 2025-03-20

**Authors:** Bosen You, Yin Sun, Jie Luo, Keliang Wang, Qing Liu, Ruizhe Fang, Bingmei Liu, Fuju Chou, Ronghao Wang, Jialin Meng, Chi-Ping Huang, Shuyuan Yeh, Chawnshang Chang, Wanhai Xu

**Affiliations:** 1 Department of Urology, The 4th Affiliated Hospital of Harbin Medical University, Harbin, 150001 China; 2 Department of Urology, The 2nd Affiliated Hospital of Harbin Medical University, Harbin, 150001 China; 3 George Whipple Lab for Cancer Research, Departments of Pathology and Urology, and The Wilmot Cancer Institute, University of Rochester Medical Center, Rochester, NY 14646 USA; 4 Department of Pathology and Cutaneous Oncology, Heilongjiang Provincial Hospital, Harbin, 150001 China; 5 Sex Hormone Research Center and Departments of Urology, China Medical University/Hospital, Taichung, 404 Taiwan

Correction to: *Oncogene* 10.1038/s41388-020-01616-1, published online 28 January 2021

Following the publication of this article, the authors noted the use of two incorrect images in Figure 7A. The raw data was reviewed and the corrected figure is provided below. The authors confirm this amendment has no impact on the results or conclusions of this study.

Former Fig. 7A:
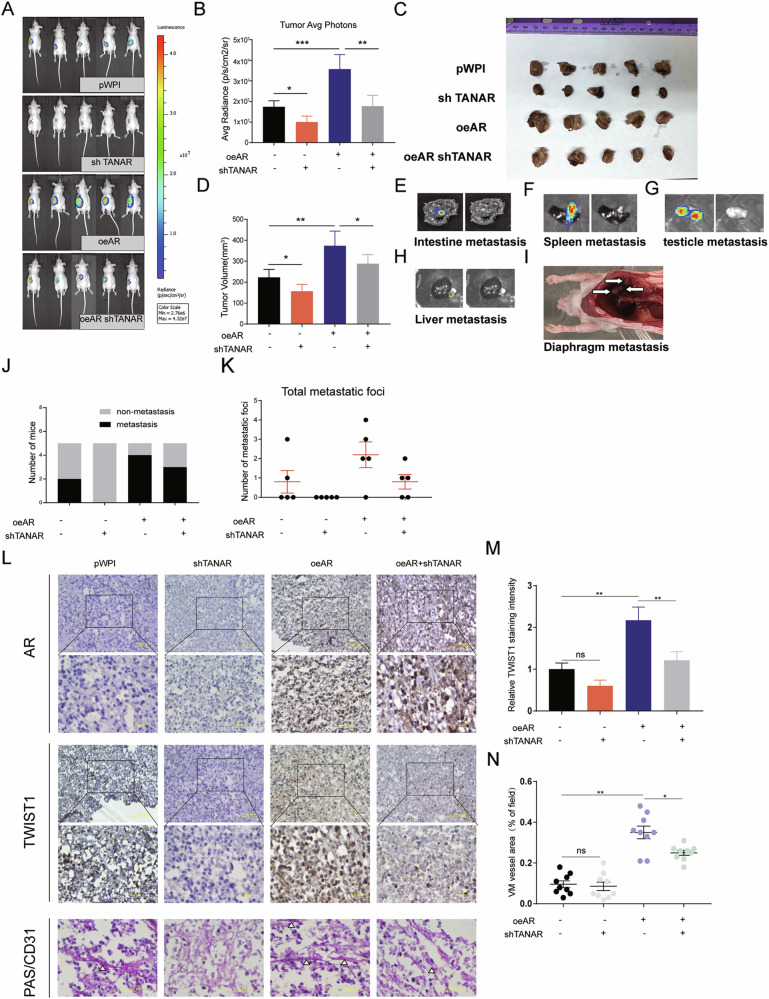


Corrected Fig 7A:
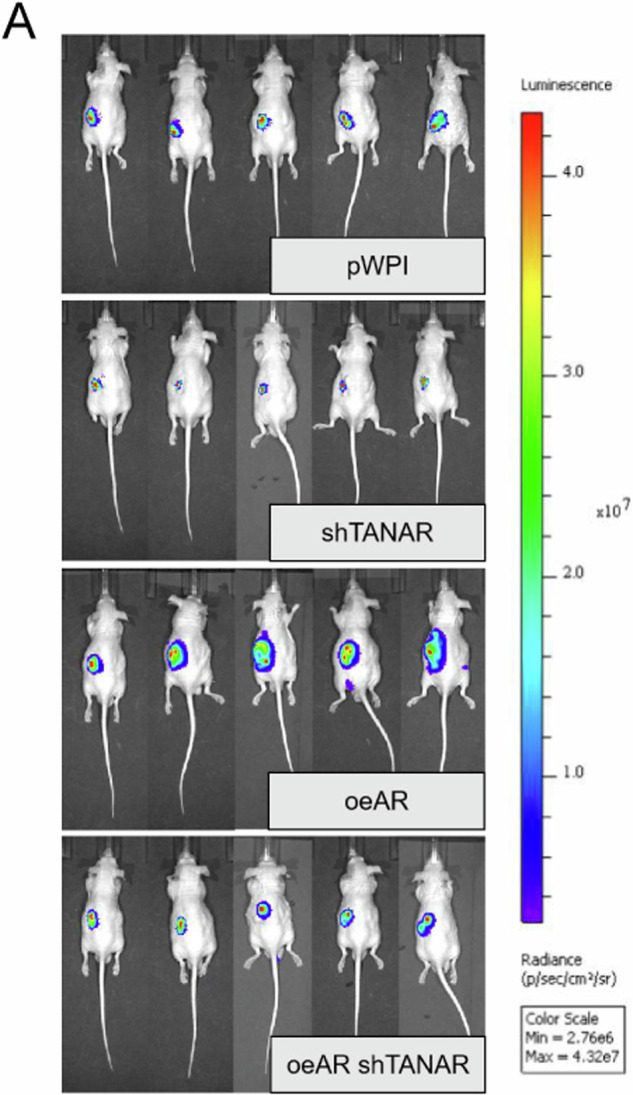


The original article has been corrected.

