# Supplementary material for: Androgen receptor promotes renal cell carcinoma (RCC) vasculogenic mimicry (VM) via altering TWIST1 nonsense-mediated decay through lncRNA-TANAR
Source: Oncogene. 2021 Jan 28;40(9):1674–89. doi: 10.1038/s41388-020-01616-1 (PMC7932923; doi:10.1038/s41388-020-01616-1)
Supplement: Supplementary file 1 — supplement figure legend [file 41388_2020_1616_MOESM1_ESM.docx]

**SFig. 1.** (A) VM channels per high-quality frame (HPF) in Male (n=36) and Female (n=15) patients samples. (B) VM formation was quantified in TWIST1-high (n = 24) and TWIST1-low (n = 27) tumor tissues. Red=VM Negative and black=VM Positive. (C) 2D & 3D VM tube formation assay for ethanol/10 nM DHT and DMSO/10 μM Enz treated SW839 and 786 cells, respectively.(D) Western blot assay for AR, and TWIST1 protein levels in OSRC-2 cells with pLKO or shAR and with pWPI or oeAR. (E) 2D Matrigel-coated VM assay were performed in OSRC-2 cells transfected as indicated. (F) 3D Collagen I-based VM assay were performed in in OSRC-2 cells transfected as indicated. (G) Western blot assays were performed on SW839 cells transfected with pLKO, shAR^1#^ or shAR^2#^. (H) 2D Matrigel-coated VM assay were performed in SW839 cells transfected as indicated. (I) 3D Matrigel-coated VM assay were performed in SW839 cells transfected as indicated.

**SFig. 2** (A) RT-PCR analyses were performed after treated 786O (left) and SW839 (right) cells with Ethanol/10 nM DHT or DMSO/10 μM Enz, respectively. (B) Ago2 pull-down assays were performed for RT-PCR. The TWIST1 mRNA levels were detected in 786O cells transfected with pWPI or oeAR. (C) The RT-PCR was applied to validate the knockdown efficiency of 5 lncRNA candidates. (D) The RT-PCR was applied to validate the overexpression efficiency of ENST 00000425110.1 and ENST 00000377977.1. (E-F) Western blot assays were performed on 786O cells (E) transfected with pWPI+pLKO,pWPI+sh ENST00000377977,oeAR+pLKO and oeAR+sh ENST00000377977 and on SW839 (F) cells transfected with pLKO+pWPI, pLKO+oe ENST00000377977, shAR+pWPI and shAR+oe ENST00000377977.（G-H）RT-PCR analyses were performed to detect TANAR expression after treated 786O (G) and SW839 (H) cells with Ethanol/10 nM DHT or DMSO/10 μM Enz, respectively.

**SFig. 3** (A) The chromosome location of TANAR based on ensemble software. (B) The protein-encoding possibility of lncRNA-TANAR predicted by LNCipedia.org. (C) The lncRNA-TANAR localization predicted by LncLocator. (D-E) Co-transfection of ARE wild type (WT) or Mutant (MT) TANAR promoter pGL3-Luciferase plasmids into SW839 cells with ethnol or DHT 10nM (D) and SW839 cells with DMSO or Enz 10uM (E).The luciferase reporter assay was performed to detect promoter activity.

**SFig. 4** (A) The potential binding site structure and joint secondary structure between TANAR and TWIST1 analyzed by online RNA-RNA predicting software (rtools.cbrc.jp). (B) The overexpression efficiency of TANAR wild type (WT) and mutant (MT) through RT-PCR. (C) Ensembl online database demonstrated the nonsense-mediated mRNA decay occurred in TWIST1 transcripts. (D) Western blot was performed to detect the TANAR impact on UPF1 protein expression. (E-F) Transfection of pWPI-luc-TWIST1, pWPI-luc-mutant 1 or pWPI-luc-mutant 2 plasmids into 786O cells with 10 nM DHT for 24 h (E) or into SW839 cells treated with 10 μM Enz for 24 h (F). The luciferase reporter assay was performed to detect promoter activity. The data are the means ± S.D. *p < 0.05, and ns=not significant, compared with control.
