# Supplementary figures and images for: Androgen receptor promotes renal cell carcinoma (RCC) vasculogenic mimicry (VM) via altering TWIST1 nonsense-mediated decay through lncRNA-TANAR
Source: Oncogene. 2021 Jan 28;40(9):1674–89. doi: 10.1038/s41388-020-01616-1 (PMC7932923; doi:10.1038/s41388-020-01616-1)

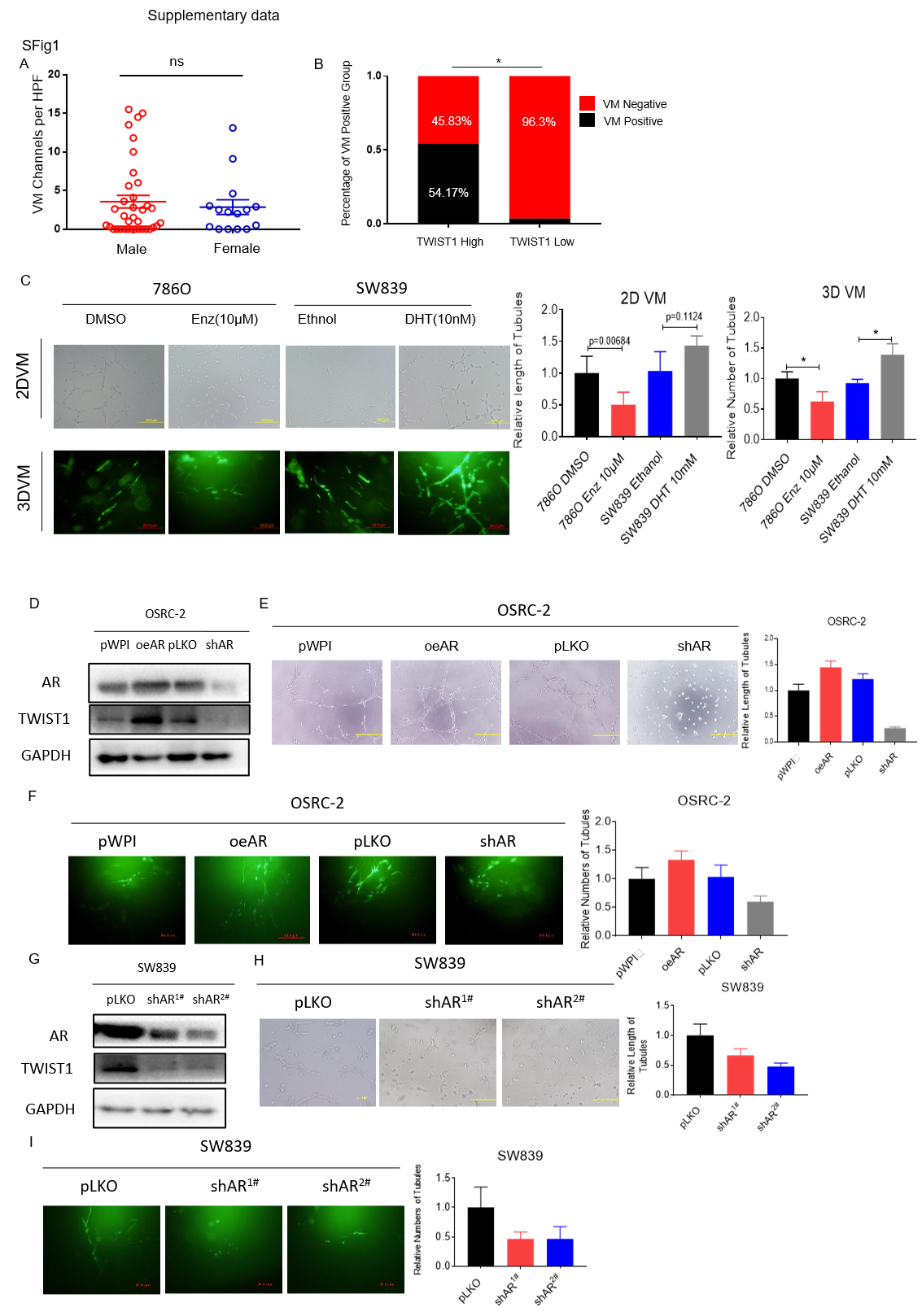

Supplement: Supplementary file 2 — supplement figure 1 [file 41388_2020_1616_MOESM2_ESM.tif]

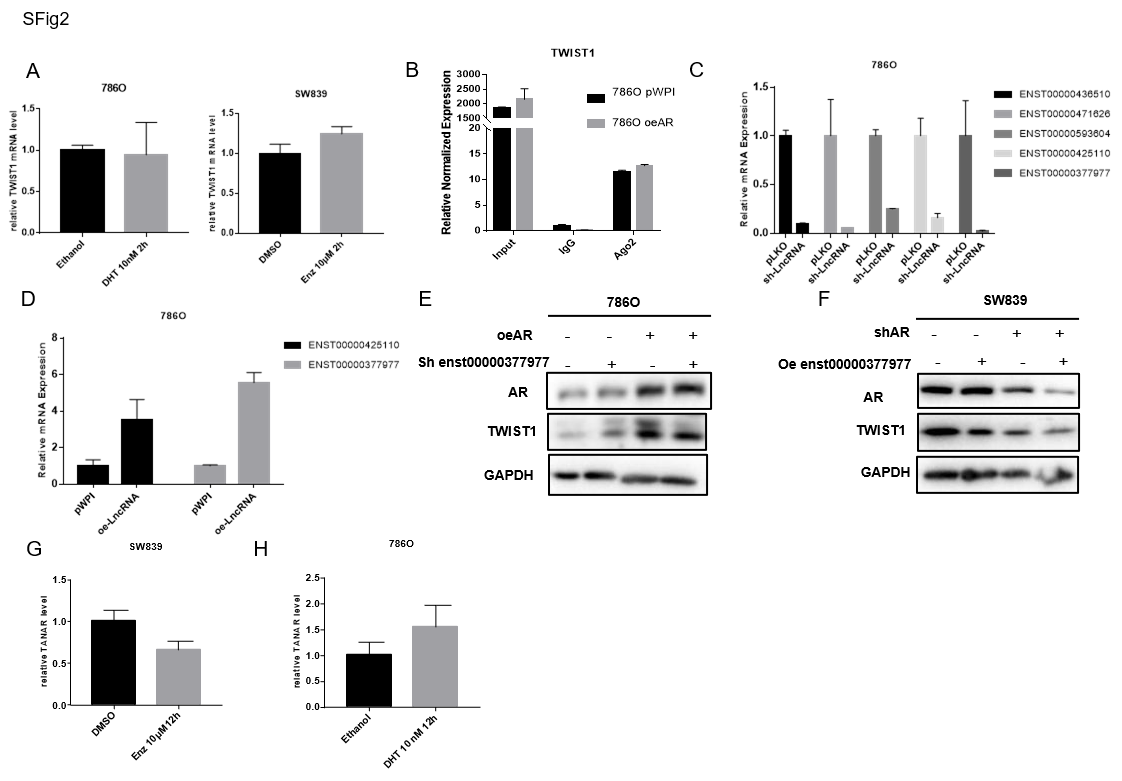

Supplement: Supplementary file 3 — supplement figure 2 [file 41388_2020_1616_MOESM3_ESM.tif]

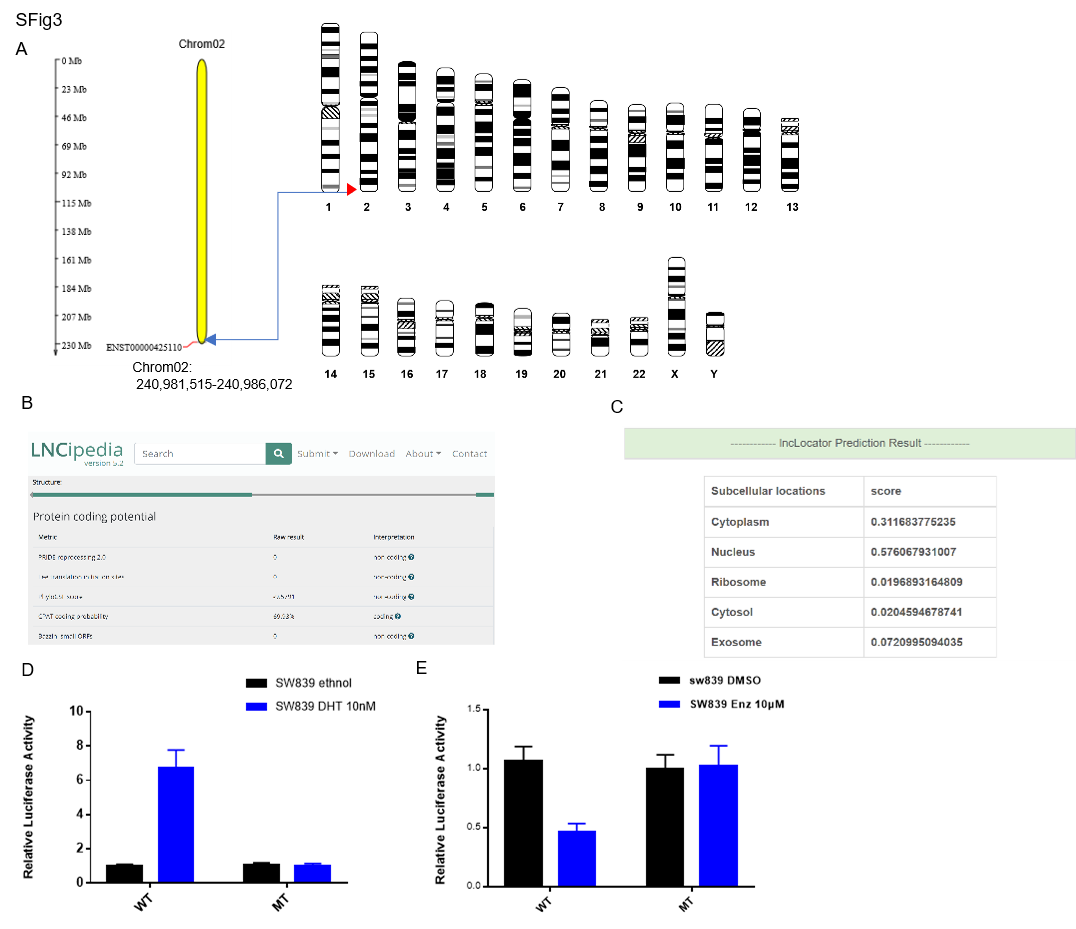

Supplement: Supplementary file 4 — supplement figure 3 [file 41388_2020_1616_MOESM4_ESM.tif]

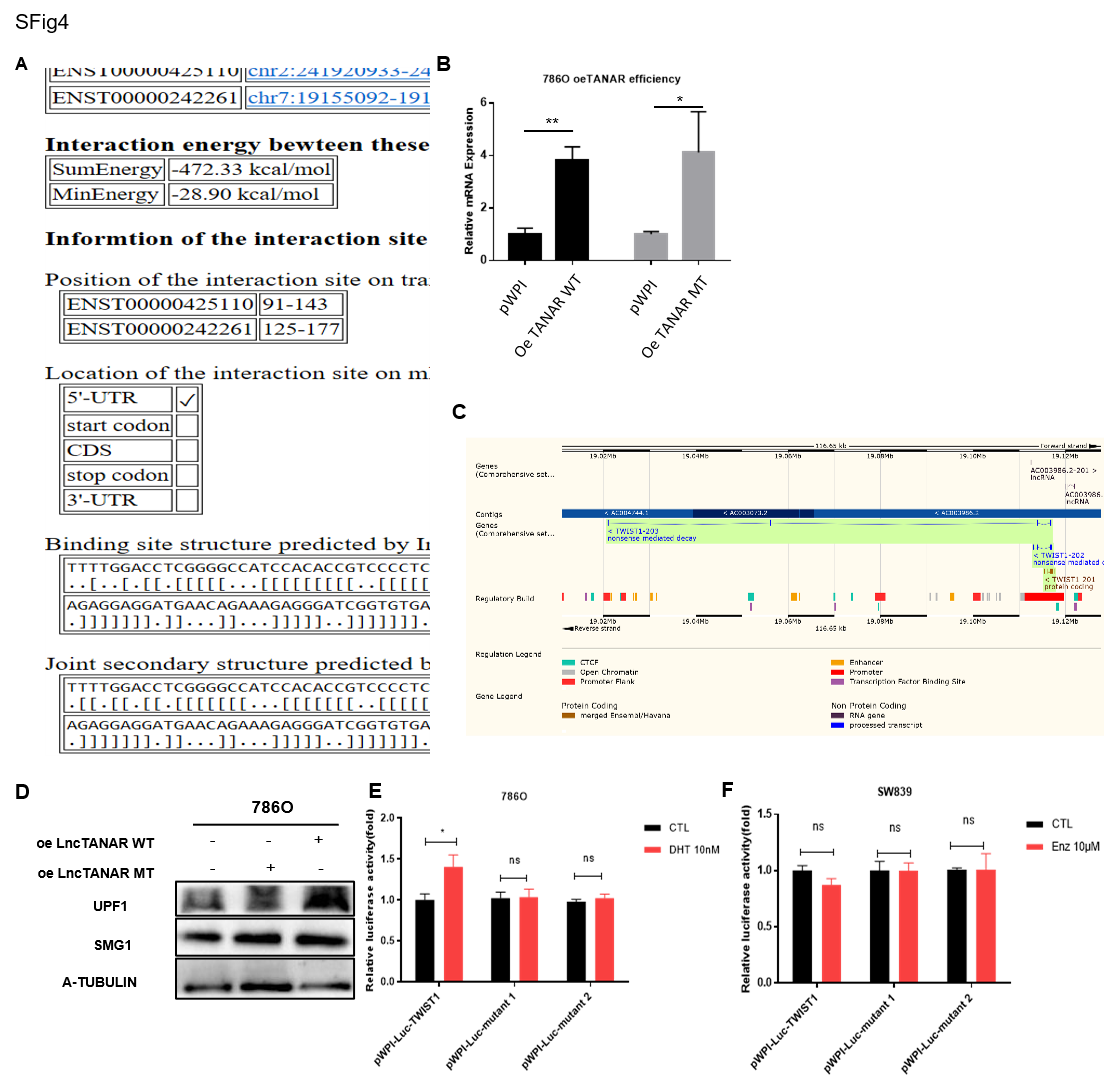

Supplement: Supplementary file 5 — supplement figure 4 [file 41388_2020_1616_MOESM5_ESM.tif]
