# Supplementary material for: Androgen receptor promotes renal cell carcinoma (RCC) vasculogenic mimicry (VM) via altering TWIST1 nonsense-mediated decay through lncRNA-TANAR
Source: Oncogene. 2021 Jan 28;40(9):1674–89. doi: 10.1038/s41388-020-01616-1 (PMC7932923; doi:10.1038/s41388-020-01616-1)
Supplement: Supplementary file 6 — Supplemental table1 [file 41388_2020_1616_MOESM6_ESM.pdf]

Supplemental table 2

<table>
<tr><th colspan="6">Upexpressed LNCRNA related to TWIST1(VM's gene)</th></tr>
<tr><th>Gene_Symbol</th><th>Transcript_Id</th><th>Log2(FC)7Su3rd/786-O</th><th>Log2(FC)ACSu3rd/ACHN</th><th>Minenergy rank</th><th>All_Gene_Info</th></tr>
<tr><td>RP11-87G24.5</td><td>ENST00000589914.1</td><td>1.855647027</td><td>2.705294125</td><td>1182</td><td>ENST00000589914.1|ENSG00000267535.1|OTTHUMG00000177703.1|OTTHUMT00000438605.1|RP11-87G24.5-002|RP11-87G24.5|457|</td></tr>
<tr><td>RP4-591L5.1</td><td>ENST00000427319.1</td><td>1.678255491</td><td>3.329689002</td><td>2296</td><td>ENST00000427319.1|ENSG00000236335.1|OTTHUMG00000003682.1|OTTHUMT00000010416.1|RP4-591L5.1-001|RP4-591L5.1|507|</td></tr>
<tr><td>AC006372.5</td><td>ENST00000449903.1</td><td>2.357850013</td><td>3.818939687</td><td>3551</td><td>ENST00000449903.1|ENSG00000223872.1|OTTHUMG00000152645.1|OTTHUMT00000327108.1|AC006372.5-001|AC006372.5|546|</td></tr>
<tr><td>RP11-973H7.4</td><td>ENST00000589930.2</td><td>1.519109343</td><td>3.157431586</td><td>4352</td><td>ENST00000589930.2|ENSG00000260302.2|OTTHUMG00000180894.2|OTTHUMT00000453525.2|RP11-973H7.4-001|RP11-973H7.4|1329|</td></tr>
<tr><td>AC159540.3</td><td>ENST00000422600.1</td><td>2.534014621</td><td>2.481636095</td><td>4783</td><td>ENST00000422600.1|ENSG00000237837.1|OTTHUMG00000152992.1|OTTHUMT00000328926.1|AC159540.3-001|AC159540.3|555|</td></tr>
<tr><td>RP11-65J3.3</td><td>ENST00000436510.1</td><td>1.930625957</td><td>3.280731705</td><td>5199</td><td>ENST00000436510.1|ENSG00000230676.1|OTTHUMG00000020780.3|OTTHUMT00000315870.1|RP11-65J3.3-002|RP11-65J3.3|1846|</td></tr>
<tr><td>RP11-475N22.2</td><td>ENST00000471626.1</td><td>1.758571096</td><td>2.761739719</td><td>5238</td><td>ENST00000471626.1|ENSG00000242049.1|OTTHUMG00000159687.1|OTTHUMT00000356923.1|RP11-475N22.2-001|RP11-475N22.2|1798|</td></tr>
<tr><td>FENDRR</td><td>ENST00000593604.1</td><td>2.044593331</td><td>2.429644606</td><td>5333</td><td>ENST00000593604.1|ENSG00000268388.5|OTTHUMG00000183870.2|OTTHUMT00000467216.1|RP11-463O9.7-007|FENDRR|960|</td></tr>
<tr><td>RP3-399L15.3</td><td>ENST00000421891.2</td><td>1.993065303</td><td>2.860557068</td><td>5416</td><td>ENST00000421891.2|ENSG00000228624.7|OTTHUMG00000164691.5|OTTHUMT00000379763.1|RP3-399L15.3-010|RP3-399L15.3|831|</td></tr>
<tr><td>AC097468.4</td><td>ENST00000441450.1</td><td>2.007802532</td><td>2.425729226</td><td>5749</td><td>ENST00000441450.1|ENSG00000224090.1|OTTHUMG00000154630.1|OTTHUMT00000336400.1|AC097468.4-001|AC097468.4|1992|</td></tr>
<tr><td>MEG3</td><td>ENST00000521812.1</td><td>1.786327</td><td>3.127481267</td><td>7162</td><td>ENST00000521812.1|ENSG00000214548.15|OTTHUMG00000029052.9|OTTHUMT00000381139.1|AL117190.2-020|MEG3|635|</td></tr>
<tr><td>AC104809.2</td><td>ENST00000425110.1</td><td>2.061885236</td><td>2.376837348</td><td>8472</td><td>ENST00000425110.1|ENSG00000223991.1|OTTHUMG00000151846.1|OTTHUMT00000324139.1|AC104809.2-001|AC104809.2|2345|</td></tr>
<tr><td>AC074391.1</td><td>ENST00000377977.3</td><td>2.348957799</td><td>2.332272475</td><td>8735</td><td>ENST00000377977.3|ENSG00000204929.12|OTTHUMG00000152768.6|OTTHUMT00000470883.1|AC074391.1-010|AC074391.1|1266|</td></tr>
<tr><td>RP11-449L23.2</td><td>ENST00000583712.2</td><td>1.800873758</td><td>3.378803744</td><td>9789</td><td>ENST00000583712.2|ENSG00000264985.2|OTTHUMG00000178451.2|OTTHUMT00000490471.1|RP11-449L23.2-003|RP11-449L23.2|4315|</td></tr>
<tr><td>RP11-134P9.3</td><td>ENST00000412772.1</td><td>2.065263815</td><td>3.214323692</td><td>9884</td><td>ENST00000412772.1|ENSG00000231507.1|OTTHUMG00000041219.1|OTTHUMT00000098768.1|RP11-134P9.3-001|RP11-134P9.3|775|</td></tr>
<tr><td>CTD-3187F8.14</td><td>ENST00000600074.1</td><td>1.992345316</td><td>2.721041387</td><td>9980</td><td>ENST00000600074.1|ENSG00000269072.1|OTTHUMG00000183198.1|OTTHUMT00000465635.1|CTD-3187F8.14-001|CTD-3187F8.14|1763|</td></tr>
<tr><td>RP5-944M2.3</td><td>ENST00000397346.7</td><td>1.893431372</td><td>3.201238061</td><td>10237</td><td>ENST00000397346.7|ENSG00000214043.7|OTTHUMG00000168469.1|OTTHUMT00000399847.1|RP5-944M2.3-001|RP5-944M2.3|2458|</td></tr>
<tr><td>MIR3142HG</td><td>ENST00000441570.1</td><td>-3.359120842</td><td>-1.967855557</td><td>7125</td><td>ENST00000441570.1|ENSG00000233237.6|OTTHUMG00000015006.3|OTTHUMT00000041166.2|RP3-331H24.3-002|LINC00472|475|</td></tr>
<tr><td>RP3-354J5.2</td><td>ENST00000534499.1</td><td>-3.966777341</td><td>-2.50413701</td><td>8556</td><td>ENST00000534499.1|ENSG00000246067.7|OTTHUMG00000167010.2|OTTHUMT00000392431.1|RP11-113K21.5-006|RP11-113K21.5|597|</td></tr>
</table>
