# Supplementary material for: Androgen receptor promotes renal cell carcinoma (RCC) vasculogenic mimicry (VM) via altering TWIST1 nonsense-mediated decay through lncRNA-TANAR
Source: Oncogene. 2021 Jan 28;40(9):1674–89. doi: 10.1038/s41388-020-01616-1 (PMC7932923; doi:10.1038/s41388-020-01616-1)
Supplement: Supplementary file 7 — Supplemental table2 [file 41388_2020_1616_MOESM7_ESM.pdf]

Supplemental tables

Supplementary Table 1. Antibodies used

Antibody	Company (catalog)
AR	Santa Cruz, #sc–7305
GAPDH	Santa Cruz, #sc-166574
B-actin	Santa Cruz, #sc-517582
Ago2	Cell Signaling Technology, #2897
TWIST1	Abclonal #A7314
VE-cadherin	Santa Cruz, #sc-9989
UPF1	Bethyl Laboratories, #A300-38A
LAMC2	Santa Cruz, #sc-25341
D2-40	ABcam(ab77845)
