# Supplementary material for: Androgen receptor promotes renal cell carcinoma (RCC) vasculogenic mimicry (VM) via altering TWIST1 nonsense-mediated decay through lncRNA-TANAR
Source: Oncogene. 2021 Jan 28;40(9):1674–89. doi: 10.1038/s41388-020-01616-1 (PMC7932923; doi:10.1038/s41388-020-01616-1)
Supplement: Supplementary file 8 — supplement method [file 41388_2020_1616_MOESM8_ESM.pdf]

## Supplementary method

**RNA Immunoprecipitation (RIP):** Native RIP was performed as previously described. We applied RIP to perform Ago2 pull-down assay and UPF1 pull-down assays. Briefly, Cells were lysed in 1 mL ice-cold RNase-free cell lysis buffer supplemented with 1 μL RNase inhibitor (M0307S, NEB; Ipswich, MA). After storage in -80ºC refrigerator for about 40 mins, the mixture was centrifuged to collect the supernatant. Then we mixed the supernatant with Protein A/G beads to preclean. Next, we incubated cell lysates with Ago2 or UPF1 antibody overnight at 4ºC. The Protein A/G beads were added to each tube and rotated for 1 h. The complex was washed 8-10 times by RIP buffer and the RNA extracted using Trizol (Invitrogen).Then, qPCR was performed according to the manufacturer's protocol.

**2D Matrigel-based tube formation assay**: After incubating at 4°C overnight, 50 μl Growth factor reduced Matrigel (BD Biosciences, USA) was added to the wells of 96-well plate evenly and incubated at 37 °C for 1.5 h. Subsequently, 100 μl cells were resuspended with serum-free DMEM and loaded onto the surface of Matrigel at $2 \times 10^4$ cells/well. After incubation at 37 °C for 6 h, tube formation was analyzed using microscopy (Olympus, Tokyo, Japan). Tubules were quantified by ImageJ software. Tubule lengths in each field were photographed and an average of tubule lengths in 3-5 random fields in each well were calculated.

**3D Collagen 1-induced tube formation assay**: After mixing 10x reconstitution buffer,1:1(v/v) with cells suspended in DMEM media, with soluble rat tail type I collagen in acetic acid (Corning, Corning, NY) to the final concentration, and 1 M NaOH was used to normalize pH (pH 7, 10–20 μl 1 M NaOH). Then 200 μl mixture was loaded in 48-well culture plates and incubated in a humidified 5%(v/v) $CO_2$ incubator at 37ºC for 7 days. Then VM formation was viewed under microscopy.

**The lncRNA/mRNA pull-down assay:** After receiving assigned treatments, the cells were harvested in cell lysis buffer. Detecting the levels of GAPDH *via* qRT-PCR guaranteed that the

input of each group had equal loading for the following procedures. The cell lysate mixtures supplemented with 1.0 μl RNase inhibitor and 500 pMole biotin-labeled anti-sense oligos against enst00000425110.1 (5'-CAG TGT CTC CAG GTG GAC CCT GTG TCT CCT-3') or TWIST1 mRNA (5''-AGC TTG CCA TCT TGG AGT CCA G-3') were rotated overnight at 4°C. The cell lysis mixtures incubated with 10 μl Streptavidin Agarose beads were rotated for 1 h at 4°C and mixtures washed by RIP buffer five times. Total RNA or protein was extracted according to the manufacturer's protocol, and qPCR analysis or Western blots were performed to detect mRNA or protein levels, respectively.

**Luciferase Reporter Assay:** 2000bp human lncRNA-TANAR(ENST00000425110.1) promoter was cloned into PGL3 basic vectors (Promega). By mutating the crucial site of AR binding site in the lncRNA-TANAR 5' promoter to EcoRI cutting site (-GAATTC), we constructed the mutant promoter PGL3 vector. PRL-TK was used as an internal control that served as the baseline control response. 786O and SW839 cells were plated in 24-well plates and the cDNA was transfected with Lipofectamine 3000 transfection reagent (Invitrogen) according to the manufacturer's instructions. Luciferase activity was measured 36-48 hrs after transfection by Dual-Luciferase Assay (Promega) according to the manufacturer's manual.

**RNA fluorescence in situ hybridization (FISH):** FISH was performed to detect the presence of lncRNA-TANAR and TWIST1 mRNA, using a designed biotin-labeled probe (VA6-16446-VC, Thermofisher Scientific, Invitrogen™). The signals of the probes were detected by ViewRNA™ ISH Cell Assay Kit (QVC0001, Thermofisher Scientific, Invitrogen™) according to the manufacturer's instructions and previous literature.The images were recorded by AxioImagerZ.1 fluorescence microscope.

**Immunofluorescence**: Immunofluorescence was performed with paraffin sections according to standard procedures. In brief, the slides were deparaffinized and hydrated followed by antigen retrieval. After being blocked with 10% goat serum, the slides were incubated with the primary antibody against AR (dilution 1:200) and LAMC2 (dilution 1:200) overnight at 4°C. Next, slides were labeled with two different secondary antibodies for 1 h at room temperature. After washing

three times with PBS, the slides were subsequently labeled with DAPI for visualization of staining intensity.

**Immunohistochemistry (IHC) staining**. After fixing in 4% neutral buffered paraformaldehyde for 18 h, the patient or mouse tissue samples were embedded in paraffin and sequentially cut into 4 μm slices. After deparaffinization, hydration, antigen retrieval, and blocking, these slices were incubated with corresponding primary antibodies, then incubated with biotinylated secondary antibodies (Vector Laboratories, Burlingame, CA, USA), and finally visualized by VECTASTAIN ABC peroxidase system and 3, 3'-diaminobenzidine (DAB) kit (Vector Laboratories). The percentage of positive cells was rated per high-power field (HPF) using 400× magnification as follows: sections with 1% positive cells were rated as 0, 1 to 25% positive cells as 1, 26 to 50% positive cells as 2, 51% to 75% positive cells as 3, and 76% to 100% positive cells as 4. The staining intensity was rated as follows: 1 for weak intensity, 2 for moderate intensity, and 3 for high intensity. Points for staining intensity and the percentage of positive cells were multiplied. Tumor specimens were classified into 3 groups according to overall scoring: negative expression as 0 to 1, weak expression as 2 to 4, and high expression as 6 to 12 points. Total scores were as follows: 0 to 4 (low) and 6 to 12 (high). All slides were evaluated independently by 2 pathologists without knowledge of the identity of patients and the clinical outcome.

**Lentivirus packaging and cell transfection:** The plasmids pLKO.1-shAR, pLKO.1-shTWIST1, pLKO.1-ENST00000425110.1 (TANAR1# and TANAR2#), pLKO.1-ENST00000436510.1, pLKO.1-ENST00000471626.1, pLKO.1-ENST00000593604.1, pLKO.1-ENST00000377977.3, pWPI-AR, pWPI-TWIST1, pWPI-TANAR wild-type, and pWPI-TANAR mutant were co-transfected with package and envelope plasmids, psPAX2 and pMD2.G into HEK293T cells for 48 h following the standard calcium phosphate transfection method to produce the lentivirus particle soup, which was then collected and stored at -80°C for later infection of ccRCC cells.

**Chromatin Immunoprecipitation Assay (ChIP)**: In brief, SW839 cells were cross-linked with 3% formaldehyde and lysed in lysis buffer, then, we extracted and sonicated lysates. Protein A/G

beads were used to preclear the chromatin. We then added anti-AR antibody (2.0 μg) or anti-IgG antibody to the DNA-protein mixture and incubated overnight at 4°C. Specific primer sets were designed to amplify a target sequence within the human ENST00000425110.1 promoter and agarose gel electrophoresis was used to identify the PCR products. The sequences of the primers are listed below:

| | left | right |
|---|---|---|
| ARE1 | CCCTCTGGAGGAAAGGATTG | GATCCCTTCCACACCAACTC |
| ARE2 | GGGGATAATTTGCATGGGTA | CATGCACTTCCAGGAGTGTAAC |
| ARE3 | GGGGATAATTTGCATGGGTA | CATGCACTTCCAGGAGTGTAAC |
| ARE4 | CCCTCTGGAGGAAAGGATTG | GATCCCTTCCACACCAACTC |
| ARE5 | TTCGTGACTGTCCTGACCTG | CATCTGTGAAGTGGGGAAGG |

**RNA extraction and quantitative real-time PCR (qRT-PCR) analysis:** Total RNAs were isolated using Trizol reagent (Invitrogen, Grand Island, NY), and 2 μg of total RNA was subjected to reverse transcription using Superscript III transcriptase (Invitrogen). Real-time PCR (RT-PCR) was conducted using a Bio-Rad CFX96 system with SYBR green to determine the mRNA expression level of a gene of interest. Expression levels were normalized to the GAPDH level using the $2^{-\Delta\Delta}$Ct methods. The sequences of the primers are listed below:

| **Transcript_Id** | **Forward primer** | **Reverse primer** |
|---|---|---|
| ENST00000589914.1 | TAAGCCACAAACAGAAGCCAG | TCCAAATTGAAGATCGCACCT |
| ENST00000427319.1 | CCATCGTTAACGGGTGGAGG | TTTTCTGTGGCATCTCGGGT |
| ENST00000449903.1 | GAACCAGGTAAACCCAGCCC | TTTAGCACGATTCCCCCAGC |
| ENST00000589930.2 | TGATGAGGTCACCAGCCCTA | TCGCTGAGCTGCGTATGAAC |
| ENST00000422600.1 | TCCACACTCCCCCAGCTTTA | CCAGCTTCCGCTGCTTTTAC |
| ENST00000436510.1 | ACCCAGGGCTCCACATCTAT | TAACCAGCTCGAACAACCCC |
| ENST00000471626.1 | ACACACCAATGTGCGAATGC | GTCACCTCTTCTGACCAGCC |
| ENST00000593604.1 | TGCATCACTGTCAAAACCAGC | AGTCCTGTTCCACCTCCGTC |
| ENST00000421891.2 | AGTAGAGTGACGGGGTGGTG | GGTTTGCTCCTCCCTCCTTTT |
| ENST00000441450.1 | AGAGGGGTGCAAAGCCATAC | AAGTGCGGGAGTCATGGAAG |
| ENST00000521812.1 | ATCCAAAGACGGTTTCCCCT | GGGTTTACCAGGATGTGGGA |
| ENST00000425110.1 | ACCGTATCGCTGGGATGAAA | CCAAGTCTTTGCCCACGCTA |
| ENST00000377977.3 | ACCGTATCGCTGGGATGAAA | CCAAGTCTTTGCCCACGCTA |
| ENST00000412772.1 | AGGAGGCGTCTATAGGGGAA | CCTGGCCTATCAGTTCTCCAC |
| ENST00000600074.1 | AGTCAACGGAGATGCTGCAA | TCAGGTCGCTCAGGTACTCA |
| ENST00000400282.6 | GAAGATGGTGGCGGCTACTC | GACAGGTCTGCCTTTTAGAGGA |
| ENST00000469070.1 | CTCTGGAAACGCCCATGAGA | GTCCTCCACTCACCTGCTTT |
| ENST00000441570.1 | TTTCTCGACTCGTCGTCAGC | ATGCCCAGTGGAGTACCTGA |

| | | |
|---|---|---|
| ENST00000534499.1 | CGGGAGGGGTGGGATATTATTG | TCCATGACCGATGTTACTCTGG |
